# Change in body weight is positively related to the change in muscle mass of the quadriceps in older inpatients with severely low BMI according to the GLIM criteria

**DOI:** 10.1186/s12877-024-05309-2

**Published:** 2024-08-26

**Authors:** Naoki Akazawa, Keita Funai, Toshikazu Hino, Ryota Tsuji, Wataru Tamura, Kimiyuki Tamura, Akemi Hioka, Hideki Moriyama

**Affiliations:** 1 Department of Physical Therapy, Faculty of Health and Welfare, Tokushima Bunri University, Hoji 180, Nishihama, Yamashiro-cho, Tokushima-city, Tokushima, 770-8514 Tokushima Japan; 2 Department of Rehabilitation, Kasei Tamura Hospital, Wakayama, Wakayama Japan; 3 Life and Medical Sciences Area, Health Sciences Discipline, Kobe University, Kobe, Hyogo Japan

**Keywords:** Body weight, Severely low BMI, GLIM, Muscle mass, Quadriceps, Older inpatients

## Abstract

**Background & aims:**

Body weight is one of the essential indicators of nutritional status, and body weight management is vital in nutritional care. In addition, low body mass index (BMI) was included as a phenotypic criterion in the Global Leadership Initiative on Malnutrition (GLIM) criteria. Furthermore, low BMI has been used in grading the severity of malnutrition (moderate or severe malnutrition) in the GLIM criteria. A recent cross-sectional study reported that muscle mass of the quadriceps in older inpatients with severely low BMI is less than those of older inpatients with moderately low BMI and non-low BMI. However, the longitudinal relationship between body weight and muscle mass of the quadriceps in older inpatients in each BMI category according to the GLIM criteria remains unclear. This study aimed to examine the longitudinal relationship between body weight and muscle mass of the quadriceps in older inpatients in each BMI category according to the GLIM criteria.

**Methods:**

This retrospective cohort study included 179 older inpatients (aged ≥ 70 years) (median [IQR] age: 84.0 [79.0–89.0]). The period of this study was between January 2017 and March 2020. In accordance with the cut-off value of a low BMI for patients aged ≥ 70 years in the Asian population according to the GLIM criteria, the participants were divided into the following three groups: the severely low BMI group (< 17.8 kg/m^2^) (*n* = 47), moderately low BMI group (≥ 17.8 to < 20.0 kg/m^2^) (*n* = 38), and non-low BMI group (≥ 20.0 kg/m^2^) (*n* = 94). The medians (IQR) of the length of hospital stay of the severely low BMI, moderately low BMI, and non-low BMI groups were 71.0 (49.0–98.0) days, 71.0 (50.0–98.0) days, and 50.5 (36.5–103.0) days, respectively. The primary outcome was a change in muscle mass of the quadriceps. The muscle mass of the quadriceps was examined using ultrasound images (i.e., quadriceps thickness). The changes in quadriceps thickness and body weight were calculated by subtracting the quadriceps thickness and body weight at admission from those values at discharge. Multiple linear regression analysis adjusting for confounding factors was used to determine whether the change in body weight was independently and significantly related to the change in quadriceps thickness in the severely low BMI, moderately low BMI, and non-low BMI groups.

**Results:**

The means (SD) of the change in quadriceps thickness of the severely low BMI group, moderately low BMI group, and non-low BMI group were 0.0 ± 0.3 cm, 0.1 ± 0.3 cm, and 0.1 ± 0.5 cm, respectively. The means of the change in body weight in those groups were 0.4 ± 2.8 kg, − 1.1 ± 2.7 kg, and − 1.3 ± 4.3 kg, respectively. In the severely low BMI group, the change in body weight (β = 0.34, *p* = 0.006) and quadriceps thickness at admission (β = −0.62, *p* < 0.001) were significantly and independently related to the change in quadriceps thickness (R^2^ = 0.645, f^2^ = 1.817, statistical power = 1.000). In the moderately low BMI and non-low BMI groups, there were no factors that were significantly and independently related to the change in quadriceps thickness.

**Conclusions:**

The results of this study suggest that change in body weight is positively related to the change in muscle mass of the quadriceps in older inpatients with severely low BMI according to the GLIM criteria. These results imply the importance of body weight management for older inpatients with severely low BMI perspective from the muscle mass of the quadriceps.

## Introduction

Body weight is one of the essential indicators of nutritional status, and body weight management is vital in nutritional care [1, 2]. In addition, a higher malnutrition rate has been confirmed in older inpatients [3, 4]. Considering them, body weight management in older inpatients with low body weight is considered to be important. In fact, recent studies reported that low body weight negatively affects the recovery of activities of daily living (ADL) in older inpatients [5, 6].

In 2018, major academic parenteral and enteral nutrition societies around the world developed the Global Leadership Initiative on Malnutrition (GLIM) criteria [1]. Low body mass index (BMI) was included as a phenotypic criterion in the GLIM criteria [1]. Furthermore, low BMI has been used in grading the severity of malnutrition (moderate or severe malnutrition) in the GLIM criteria [1]. In 2019, Maeda et al. [7] determined the cut-off value for a low BMI to grade the malnutrition severity in the GLIM criteria in Asian populations. The authors reported that the cut-off values for moderately or severely low BMI in Asian patients aged < 70 and ≥ 70 years were 17.0 and 17.8 kg/m^2^, respectively. A recent cross-sectional study [8] reported that muscle mass of the quadriceps in older inpatients with severely low BMI is less than those of older inpatients with moderately low BMI and non-low BMI and the validity of 17.8 kg/m^2^ as the cut-off value for moderately or severely low BMI in Asian populations aged ≥ 70 years according to the GLIM criteria from the perspective of muscle mass of the quadriceps. Muscle mass of the quadriceps in older persons has been reported to be related to muscle strength [9], gait ability [10], and mortality rate [11, 12]. Furthermore, loss of muscle mass with aging and disuse particularly occurs in the quadriceps among the muscles of the upper and lower extremities [13, 14]. Based on these findings, targeting the muscle mass of the quadriceps is pivotal. However, the longitudinal relationship between body weight and muscle mass of the quadriceps in older inpatients in each BMI category according to the GLIM criteria remains unclear. Revealing them is considered to be essential for deeply understanding the importance of managing body weight in older inpatients in each BMI category. This study aimed to examine the longitudinal relationship between body weight and muscle mass of the quadriceps in older inpatients in each BMI category according to the GLIM criteria.

## Materials and methods

### Study design and participants

The inclusion criteria in this retrospective cohort study were older patients (aged ≥ 70 years) who were referred to the Department of Rehabilitation at Kasei Tamura Hospital between January 2017 and March 2020. This hospital has subacute and convalescent rehabilitation wards. Patients who died during a hospital stay, who underwent thigh amputation, and who had a lack of data were excluded. A total of 240 inpatients were included in this study. Of these, patients aged < 70 years (*n* = 55) or had lacked necessary data (*n* = 6) were excluded. Ultimately, 179 older inpatients (median [IQR] age: 84.0 [79.0–89.0]) were included in this study. The participants were found to have the following diseases: stroke (cerebral hemorrhage [*n* = 11] and cerebral infarction [*n* = 16]), fracture (hip fracture [*n* = 31], compression fracture [*n* = 27], pubic fracture [*n* = 3], and other fracture [*n* = 8]), pneumonia [*n* = 29], and others (heart disease [*n* = 8], chronic obstructive pulmonary disease [*n* = 2], cancer [*n* = 6], kidney failure [*n* = 2], dehydration [*n* = 6], urinary tract infection [*n* = 2], spinal cord disease [*n* = 7], and others [*n* = 21]). In accordance with the cut-off value of a low BMI for patients aged ≥ 70 years in the Asian population according to the GLIM criteria as reported by Maeda et al. [7], the participants were divided into the following three groups: the severely low BMI group (< 17.8 kg/m^2^) (*n* = 47), moderately low BMI group (≥ 17.8 to < 20.0 kg/m^2^) (*n* = 38), and non-low BMI group (≥ 20.0 kg/m^2^) (*n* = 94). The categorization of obesity has not been set in the GLIM criteria [1]. Therefore, although 16 patients with a BMI of 25 or higher were observed in this study, we included these patients in the non-low BMI group. Rehabilitation therapy, including physical therapy, occupational therapy, and speech and swallowing therapy, was administered to all participants. Nutrition management (i.e., adjustments of nutritional intake) was tailored to each participant based on disease conditions and changes in times and load in rehabilitation therapy.

### Outcome measures

The primary outcome was a change in muscle mass of the quadriceps. The muscle mass of the quadriceps was examined using ultrasound images (i.e., quadriceps thickness). We also measured other characteristics, including body weight, underlying disease, age, sex, height, BMI, days from disease onset, length of hospital stay, subcutaneous fat mass of the thigh, energy intake, nutritional, swallowing, and inflammatory statuses, comorbidities, number of medications, and number of units of rehabilitation therapy (1 unit of rehabilitation therapy = 20 min), and ADL. Quadriceps thickness, body weight, subcutaneous fat mass of the thigh, energy intake, number of units of rehabilitation therapy, and ADL were measured not only at admission but also at discharge. The changes in quadriceps thickness and body weight were calculated by subtracting the quadriceps thickness and body weight at admission from those values at discharge. The length of hospital stay (days) and days from disease onset were assessed at discharge. The length of hospital stay was evaluated based on the hospitalization period at Kasei Tamura Hospital. Most inpatients were initially admitted to other acute-phase hospitals. Therefore, the lengths of both hospital stays were summed up and used as the number of days from the onset of the disease.

### Measurements of muscle mass in the quadriceps and subcutaneous fat mass of the thigh

Transverse ultrasound images were obtained using a B-mode ultrasound system (NanoMaxx; SonoSite Japan, Tokyo, Japan) with a linear array probe (L25n/13–6 MHz; NanoMaxx). The muscle mass of the rectus femoris and vastus intermedius of all participants were evaluated based on muscle thickness [8–10, 15, 16]. The validity of muscle mass measurement using ultrasound has been confirmed in a previous study using magnetic resonance imaging [17]. Images of the rectus femoris and vastus intermedius were obtained at 30% of the distance from the anterior superior iliac spine to the proximal end of the patella [8–10, 15, 16]. The participants laid in a supine position with their lower limbs relaxed while a water-soluble transmission gel was applied to the skin surface of the thigh. The probe was pressed lightly against the skin to prevent muscle deformation. All the ultrasound images were captured by the same investigator. The thickness of the rectus femoris was determined as the distance between the superficial adipose tissue–muscle interface and the deep muscle–muscle interface [8–10, 15, 16], while that of the vastus intermedius was determined as the distance between the superficial muscle–muscle interface and the bone–muscle interface [8–10, 15, 16]. Muscle thickness was measured using the ImageJ 1.49 software (National Institutes of Health, Bethesda, MD, USA) [8–10, 15, 16].

The sum of the thicknesses of the rectus femoris and vastus intermedius was used as quadriceps thickness. The mean quadriceps thickness on the right and left sides was included in the analysis. Measurements of the rectus femoris and vastus intermedius muscle thicknesses have relatively high reliability (intraclass correlation coefficients [1.1], 0.897–0.959) [10]. Subcutaneous fat mass in the thigh was assessed based on the subcutaneous fat thickness, which was defined as the distance between the dermis and adipose tissue interface and the muscle–adipose tissue interface [8–10, 15, 16]. The mean subcutaneous fat thickness of the right and left thighs was used in the analysis.

### BMI measurement

The BMI was calculated by dividing body weight (kg) by height squared (m^2^) [8]. Body weight was measured with a digital scale in the standing or wheelchair-sitting position in accordance with the participants’ condition and was recorded to the nearest 0.1 kg [8]. Height was measured in the standing position using a stadiometer and was recorded to the nearest 0.5 cm [8]. For patients who could not stand on the stadiometer, we measured height using a tape measure in the supine position [8]. Body weight and height were measured by a trained physical therapist, occupational therapist, nurse, or medical technologist [8].

### Measures of other characteristics

A nurse or dietitian estimated the energy intake based on a visual assessment of the intake ratio against the provided meal to the patient. The energy intake (kcal/ideal body weight [IBW] /day) was calculated based on 1 week after admission and 1 week before discharge. IBW was calculated using the following formula: IBW = height^2^ (m^2^) × 22 [18]. Malnutrition risk was assessed using the Geriatric Nutritional Risk Index (GNRI) score. The GNRI score was calculated using the following formula: GNRI score = (14.89 × serum albumin [g/dL]) + (41.7 × body weight/IBW) [19]. Swallowing ability was assessed using the Food Intake Level Scale (FILS) [20]. The FILS is a 10-point observer-rated scale, with higher values indicating better swallowing ability. The inflammatory status was assessed by analyzing C-reactive protein (CRP) concentration. A blood sample of each participant was sent to the clinical laboratory and stored at 20 °C. Approximately 3 h after sampling, CRP-Latex X2 Seiken reagent kit (Denka-Seiken, Tokyo, Japan) and Ci16000 analysis equipment (Canon, Tokyo, Japan) were used for analyzing CRP. Serum albumin was evaluated with the Accuras auto ALB II reagent kit (Shino-Test, Tokyo, Japan) and Ci16000 analysis equipment. Comorbidities were evaluated using the updated Charlson Comorbidity Index (UCCI) [21]. ADL was assessed using the Barthel Index (BI) [22]. The BI is widely used in clinical settings and includes ordinal assessment (0–100 points) [22]. Lower BI scores indicate poor ability to perform ADL.

### Statistical analysis

All statistical analyses were conducted using SPSS version 28 software (IBM SPSS Japan, Tokyo, Japan). Variables were assessed for normality using the Shapiro-Wilk test. Parametric data are reported as mean ± standard deviation, whereas nonparametric data are expressed as median (interquartile range [IQR]). Categorical data are presented as numbers (%).

Quadriceps thickness, body weight, and subcutaneous fat thickness of the thigh at admission were compared between the severely low BMI, moderately low BMI, and non-low BMI groups using analysis of covariance adjusting for age and sex. Analysis of variance was used for comparing GNRI score and serum albumin at admission between the severely low BMI, moderately low BMI, and non-low BMI groups. Differences in age, height, FILS, energy intake, UCCI, number of medications, CRP, number of units of rehabilitation therapy, and BI score at admission between the severely low BMI, moderately low BMI, and non-low BMI groups were assessed with the Kruskal–Wallis test. If the main effect was confirmed in the analysis of covariance, analysis of variance, and Kruskal–Wallis test, Bonferroni’s post hoc test was used to determine the significance. The chi-square test was used to examine the significant differences among the three groups in terms of sex and disease. A paired t-test was used to compare the quadriceps thickness and body weight in the severely low BMI, moderately low BMI, and non-low BMI groups at admission and discharge. The energy intake, subcutaneous fat thickness of the thigh, number of units of rehabilitation therapy, and BI score in the three groups at admission and discharge were compared using the Wilcoxon signed-rank test. In addition, to determine the presence or absence of interaction between measurement time (i.e., at admission and discharge) and group (i.e., the severely low BMI, moderately low BMI, and non-low BMI groups) factors, we conducted the analysis of variance for split-plot factorial design for quadriceps thickness, body weight, energy intake, subcutaneous fat thickness of the thigh, number of rehabilitation therapy, and BI score.

The associations of change in body weight with change in quadriceps thickness in the severely low BMI, moderately low BMI, and non-low BMI groups were determined using Pearson’s correlation coefficient. Multiple linear regression analysis (forced entry method) was used to determine whether the change in body weight was independently and significantly related to the change in quadriceps thickness in the severely low BMI, moderately low BMI, and non-low BMI groups. The independent variables were age, sex (male = 1, female = 0), days from disease onset, disease (stroke, fracture, pneumonia, and others: reference = stroke), quadriceps thickness at admission, and change in body weight. Recent studies [23–25] reported that changes in body weight and quadriceps thickness commonly occur in convalescent stroke patients. Based on these findings, stroke was used as the reference in the disease of this study. In addition, energy intake and FILS at admission were also included as independent variables because energy intake and swallowing ability have been reported to be related to quadriceps thickness [26, 27]. The variance inflation factor was used to assess multicollinearity; a value > 10 was considered indicative of the presence of multicollinearity. *P* < 0.05 was considered to express statistical significance. We calculated the effect size (f^2^) of the multiple linear regression analysis for change in quadriceps thickness using the following equation: R^2^/ (1 − R^2^). The statistical power of the analysis was calculated using G* Power version 3.1.9.2, based on f^2^, an alpha error of 0.05, the total sample size, and a number of predictor variables.

### Sample size calculation

A previous cross-sectional study [15] reported that the effect size (f^2^) of the multiple linear regression analysis for examining the relationship between BMI and quadriceps thickness of chronic stroke patients was 0.59. We expected to observe a similar effect size in the multiple linear regression analyses for the relationship between changes in quadriceps thickness and body weight in this longitudinal study. A priori sample size calculation with an effect size (f^2^) of 0.59, power of 0.80, alpha error of 0.05, and a number of predictors of 9 indicated that a sample size of at least 36 participants was required in each BMI group. Sample size calculations were conducted using G* Power version 3.1.9.2 (Heinrich-Heine-Universität Düsseldorf, Düsseldorf, Germany).

## Results

The medians (IQR) of the BMI in the severely low BMI, moderately low BMI, and non-low BMI groups were 15.9 (14.7–16.8) kg/m^2^, 18.8 (18.3–19.4) kg/m^2^, and 22.7 (21.2–24.1) kg/m^2^, respectively. The medians (IQR) of the day from disease onset of the severely low BMI, moderately low BMI, and non-low BMI groups were 94.0 (66.0–11.0) days, 77.0 (53.8-134.5) days, and 90.0 (64.8-117.5) days, respectively. The medians (IQR) of the length of hospital stay of the severely low BMI, moderately low BMI, and non-low BMI groups were 71.0 (49.0–98.0) days, 71.0 (50.0–98.0) days, and 50.5 (36.5–103.0) days, respectively.

Table 1 shows the characteristics of the total participants and the severely low BMI, moderately low BMI, and non-low BMI groups at admission and the results of comparisons in the characteristics among the three groups. There were main effects of quadriceps thickness, body weight, subcutaneous fat thicknesses of the thigh, GNRI score, FILS, and number of medications. Quadriceps thickness, body weight, GNRI score, and subcutaneous fat thicknesses of the thigh of the severely low BMI group were significantly lower than those of the moderately low and non-low BMI groups. In addition, these variables of the moderately low BMI group were significantly lower than the non-low BMI group. Significantly lower FILS was observed in the severely low BMI group compared to the non-low BMI group. The number of medications in the severely low BMI and moderately low BMI groups was significantly lower than that of the non-low BMI group. No significant differences were observed for the other comparisons. Table 2 shows the results of comparison analysis for the quadriceps thickness, body weight, energy intake, subcutaneous fat thickness of the thigh, number of units of rehabilitation therapy, and BI score at admission and discharge in the three groups. In the severely low BMI group, no significant differences were observed in quadriceps thickness and body weight. The subcutaneous fat thickness of the thigh, energy intake, number of units of rehabilitation therapy, and BI score at discharge were significantly higher than at admission. In the moderately low BMI group, body weight at discharge was significantly lower than at admission. Significantly higher energy intake, number of units of rehabilitation therapy, and BI score at discharge were observed compared to admission. There were no significant differences in quadriceps thickness and subcutaneous fat thickness of the thigh. In the non-low BMI group, quadriceps thickness, energy intake, units of rehabilitation therapy, and BI score at discharge were significantly higher than at admission. Significantly lower body weight at discharge was confirmed compared to admission. No significant difference was observed in the subcutaneous fat thickness of the thigh. The results of the analysis of variance for split-plot factorial design, significant main effects for measurement time factor were observed in body weight, energy intake, subcutaneous fat thickness of the thigh, number of rehabilitation therapy, and BI score. There was no main effect of the measurement time factor on quadriceps thickness. A significant interaction between measurement time and group factors was observed in body weight. There were no interactions between measurement time and group factors in quadriceps thickness, energy intake, subcutaneous fat thickness of the thigh, number of rehabilitation therapy, and BI score.

**Table 1:** Characteristics of the total participants and the severely low BMI, moderately low BMI, and non-low BMI groups at admission and the results of comparisons in the characteristics among the three groups

	Total	Severely low BMI group	Moderately low BMI group	Non-low BMI group	
	(*n* = 179)	(*n* = 47)	(*n* = 38)	(*n* = 94)	Main effect *p*-value
Age, years	84.0 (79.0–89.0)	85.0 (80.0–89.0)	84.0 (78.0–88.0)	84.0 (78.0–88.0)	0.501^a^
Sex, male/female	75 (41.9)/104 (58.1)	16 (34.0)/31 (66.0)	19 (50.0)/19 (50.0)	40 (42.6)/54 (57.4)	0.327^b^
Height, cm	150.0 (146.0–160.0)	150.0 (145.0–160.0)	154.0 (144.8–163.0)	150.0 (147.8–158.3)	0.416^a^
Body weight, kg	47.2 ± 10.5	35.8 ± 6.0^*^	44.9 ± 5.5^**^	53.9 ± 8.3	< 0.001^c^
Disease					0.497^b^
Stroke	27 (15.1)	8 (17.0)	5 (13.2)	14 (14.9)	
Fracture	75 (41.9)	17 (36.2)	14 (36.8)	44 (46.8)	
Pneumonia	28 (15.6)	11 (23.4)	7 (18.4)	10 (10.6)	
Others	49 (27.4)	11 (23.4)	12 (31.6)	26 (27.7)	
Quadriceps thickness, cm	1.2 ± 0.4	0.8 ± 0.3^*^	1.1 ± 0.3^**^	1.4 ± 0.4	< 0.001^c^
Subcutaneous fat thickness of the thigh, cm	0.4 (0.2–0.5)	0.3 (0.2–0.3)^*^	0.3 (0.3–0.5)^**^	0.5 (0.3–0.6)	< 0.001^c^
Food Intake Level Scale	8.0 (7.0–8.0)	7.0 (7.0–8.0)^**^	8.0 (6.8–8.0)	8.0 (7.0–8.0)	0.005^a^
Energy intake, kcal/ideal body weight/day	25.4 (20.0–30.2)	24.4 (21.0–27.8)	26.6 (20.4–30.5)	26.3 (19.4–30.1)	0.607^a^
C-reactive protein, mg/dL	0.5 (0.4–1.5)	0.5 (0.4–1.9)	0.4 (0.4–2.4)	0.6 (0.4–1.2)	0.978^a^
Serum albumin, g/dL	3.3 ± 0.5	3.3 ± 0.5	3.3 ± 0.6	3.4 ± 0.5	0.324^d^
Geriatric Nutritional Risk Index score	86.6 ± 9.8	78.0 ± 7.9^*^	84.9 ± 8.6^**^	91.5 ± 7.9	< 0.001^d^
Updated Charlson comorbidity index	2.0 (0.0–3.0)	2.0 (1.0–3.0)	2.0 (0.0–4.0)	2.0 (0.0–3.0)	0.289^a^
Number of medications	7.0 (5.0–9.0)	6.0 (4.0–8.0)^**^	5.5 (3.0–8.3)^**^	8.0 (6.0–11.0)	< 0.001^a^
Number of rehabilitation therapy, units/day	3.0 (2.0–4.0)	3.0 (2.0–4.0)	3.5 (2.0–4.3)	3.0 (2.0–4.0)	0.201^a^
Barthel Index score	40.0 (20.0–60.0)	35.0 (10.0–50.0)	35.0 (20.0–61.3)	42.5 (25.0–60.0)	0.062^a^

Data are presented as median (interquartile range), n (%), or mean ± standard deviation.

BMI, body mass index.

^a^Kruskal–Wallis test; ^b^Chi-square test; ^c^Analysis of covariance adjusted for age and sex; ^d^Analysis of variance

^*^p < 0.05 (significantly different from the moderately low BMI and non-low BMI groups; based on Bonferroni’s test)

^**^p < 0.05 (significantly different from the non-low BMI group; based on Bonferroni’s test)

**Table 2:** Comparisons for the quadriceps thickness, body weight, energy intake, subcutaneous fat thickness of the thigh, number of units of rehabilitation therapy, and Barthel Index score at admission and discharge in the severely low BMI, moderately low BMI, and non-low BMI groups

	Severely low BMI group (*n* = 47)				Moderately low BMI group (*n* = 38)				Non-low BMI group (*n* = 94)				Main effect (measurement time)	Interaction (measurement time by group) *p*-value
	At admission	At discharge	*p*-value		At admission	At discharge	*p*-value		At admission	At discharge	*p*-value			
Quadriceps thickness, cm	0.8 ± 0.3	0.9 ± 0.3	0.847^a^		1.1 ± 0.3	1.2 ± 0.4	0.150^a^		1.4 ± 0.4	1.5 ± 0.7	0.033^a^		0.062^c^	0.370^c^
Body weight, kg	35.8 ± 6.0	36.2 ± 6.5	0.330^a^		44.9 ± 5.5	43.8 ± 5.5	0.018^a^		53.9 ± 8.3	52.5 ± 8.6	0.003^a^		0.023^c^	0.026^c^
Energy intake, kcal/ideal body weight/day	24.4 (21.0–27.8)	26.4 (23.6–30.1)	0.006^b^		26.6 (20.4–30.5)	28.5 (23.6–33.8)	0.008^b^		26.3 (19.4–30.1)	28.7 (24.0–32.0)	< 0.001^b^		< 0.001^c^	0.954^c^
Subcutaneous fat thickness of the thigh, cm	0.3 (0.2–0.3)	0.3 (0.2–0.4)	0.011^b^		0.3 (0.3–0.5)	0.4 (0.3–0.5)	0.158^b^		0.5 (0.3–0.6)	0.5 (0.3–0.7)	0.398^b^		0.041^c^	0.873^c^
Number of rehabilitation therapy, units/day	3.0 (2.0–4.0)	5.0 (3.0–6.0)	< 0.001^b^		3.5 (2.0–4.3)	5.0 (4.0–6.0)	< 0.001^b^		3.0 (2.0–4.0)	5.0 (4.0–6.0)	< 0.001^b^		< 0.001^c^	0.158^c^
Barthel Index score	35.0 (10.0–50.0)	45.0 (15.0–85.0)	< 0.001^b^		35.0 (20.0–61.3)	70.0 (45.0–85.0)	< 0.001^b^		42.5 (25.0–60.0)	65.0 (45.0–80.0)	< 0.001^b^		< 0.001^c^	0.322^c^

Data are presented as mean ± standard deviation and median (interquartile range).

BMI, body mass index.

^a^ Paired t-test; ^b^ Wilcoxon signed-rank test; ^c^ analysis of variance for split-plot factorial design

**Fig. 1:**
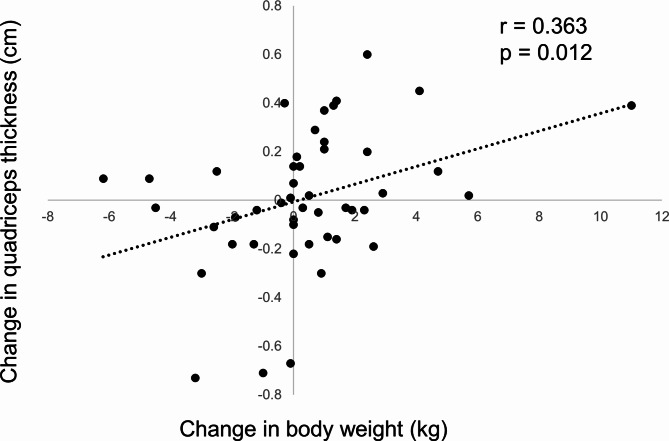
Scatter plots between the changes in quadriceps thickness and body weight in the severely low body mass index group

Table 3 shows changes in quadriceps thickness and body weight and the results of the correlation analysis for the relationships between them in the three groups. There was a positive relationship between changes in quadriceps thickness and body weight in the severely low BMI group (*r* = 0.363, *p* = 0.012). Figure 1 shows the scatter plots between the changes in quadriceps thickness and body weight in the severely low BMI group. No significant relationships were observed between changes in quadriceps thickness and body weight in the moderately low BMI (*r* = 0.041, *p* = 0.805) and non-low BMI (*r* = 0.081, *p* = 0.439) groups. Table 4 shows the results of multiple linear regression analyses which set the change in quadriceps thickness as a dependent variable in the three groups. In the severely low BMI group, the change in body weight and quadriceps thickness at admission were significantly and independently related to the change in quadriceps thickness (R^2^ = 0.645, f^2^ = 1.817, statistical power = 1.000). In the moderately low BMI and non-low BMI groups, there were no factors that were significantly and independently related to change in quadriceps thickness. Multicollinearity was not observed between the independent variables in all multiple linear regression analyses.

**Table 3:** Changes in quadriceps thickness and body weight and the results of the correlation analysis for the relationships between them in the severely low BMI, moderately low BMI, and non-low BMI groups

	Change in quadriceps thickness (cm)	Change in body weight (kg)	Pearson’s correlation coefficient	*p*-value
Severely low BMI group	0.0 ± 0.3	0.4 ± 2.8	0.363	0.012
Moderately low BMI group	0.1 ± 0.3	−1.1 ± 2.7	0.041	0.805
Non-low BMI group	0.1 ± 0.5	−1.3 ± 4.3	0.081	0.439

Data are presented as mean ± standard deviation.

BMI, body mass index.

**Table 4:** Multiple linear regression analysis for change in quadriceps thickness in the severely low BMI, moderately low BMI, and non-low BMI groups

	Change in quadriceps thickness							
	Severely low BMI group			Moderately low BMI group			Non-low BMI group	
	β	*p*-value		β	*p*-value		β	*p*-value
Age	−0.056	0.626		0.097	0.634		−0.004	0.972
Sex	−0.008	0.946		−0.311	0.167		−0.008	0.949
Days from onset disease	−0.069	0.564		0.350	0.155		0.048	0.691
Disease								
Stroke	(Reference)	-		(Reference)	-		(Reference)	-
Fracture	0.141	0.352		−0.584	0.083		−0.120	0.490
Pneumonia	−0.193	0.194		−0.358	0.196		−0.065	0.630
Others	−0.231	0.131		−0.502	0.083		−0.062	0.702
Quadriceps thickness at admission	−0.617	< 0.001		−0.197	0.294		0.059	0.625
Food Intake Level Scale at admission	0.178	0.111		0.113	0.596		0.111	0.359
Energy intake at admission	−0.165	0.122		−0.282	0.187		0.186	0.106
Change in body weight	0.339	0.006		0.189	0.325		0.030	0.792

β, standardized partial regression coefficient; BMI, body mass index

## Discussion

This study examined the longitudinal relationship between body weight and muscle mass of the quadriceps in older inpatients in each BMI category according to the GLIM criteria. Our results suggest that change in body weight is positively related to the change in muscle mass of the quadriceps in older inpatients with severely low BMI. In addition, these relationships were also confirmed after adjusting for confounding factors. These results imply the importance of body weight management for older inpatients with severely low BMI perspective from the muscle mass of the quadriceps.

Cross-sectional [15] and longitudinal [16] studies that targeted chronic stroke survivors also reported that there is a positive relationship between body weight and muscle mass of the quadriceps. However, whether a change in body weight is related to a change in muscle mass of the quadriceps in older inpatients in each BMI category according to the GLIM criteria remained unclear. Although the body weight in the severely low BMI group did not change during the hospital stay, considering the results of the correlation and multiple linear regression analysis, our results suggest that change in body weight is positively related to the change in muscle mass of the quadriceps in older inpatients with severely low BMI. Body weight loss in older inpatients with severely low BMI may be related to the loss of muscle mass in the quadriceps. This finding is important for conducting nutritional care for older inpatients with severely low BMI who especially need body weight management.

Although we targeted the severely low BMI group with no body weight change during the hospital stay, our results indicate the possibility that loss of body weight may lead to loss of muscle mass of the quadriceps in older inpatients with severely low BMI. Having this perspective is important for nutritional care in older inpatients with severely low BMI. Loss of muscle mass of the quadriceps has been reported to be related to a decrease in muscle strength [9] and gait ability [10] and an increase in mortality rate [11, 12] in older persons. Increasing body weight and protecting loss of body weight is essential for improving prognosis in older inpatients with severely low BMI.

The results of multiple linear regression analyses, the changes in body weight in older inpatients with moderately low BMI and non-low BMI were not related to the change in muscle mass of the quadriceps. Changes in body weight in older inpatients with moderately low BMI and non-low BMI may be related to changes in muscle mass and adipose tissue of another part (e.g., upper extremities or trunk) [28]. In addition, although the body weight did not change in the severely low BMI group, the body weight in the moderately low BMI and non-low BMI groups significantly decreased during a hospital stay (there was a significant interaction in body weight between measurement time and group factors). This may be also related to the results of our study. If observing an increase in body weight in the moderately low BMI and non-low BMI groups, different results may be obtained.

Recently, a possible contribution of ultrasound in the sarcopenia diagnosis has been suggested [29]. In addition, a novel diagnostic algorithm for sarcopenia suggested by the International Society of Physical and Rehabilitation Medicine has adopted muscle thickness of the quadriceps as an indicator of muscle mass [30]. Our study revealed the longitudinal relationship between the body weight and muscle mass of the quadriceps in older inpatients with severely low BMI. The finding of our study indicates the importance of monitoring for muscle thickness of the quadriceps in older inpatients with severely low BMI in body weight management. Taken together, muscle mass measurement of the quadriceps using ultrasound is considered to be important not only in sarcopenia diagnosis but also in nutritional care.

Effective energy intake for improving swallowing ability in sarcopenic dysphagia has been reported to be 35 kcal/IBW/day [31–34]. In our study, the muscle mass of the quadriceps in the severely low BMI group was significantly lower than those of the moderately low BMI and non-low BMI groups. In addition, many older inpatients who had decreased swallowing ability at admission (median [IQR] of the FILS = 7.0 [7.0–8.0]) were observed in the severely low BMI group. Based on them, the required energy intake in older inpatients with severely low BMI in this study is considered to be similar to the aforementioned energy intake (i.e., 35 kcal/IBW/day) which is effective for improving swallowing ability in sarcopenic dysphagia [31–34]. However, median (IQR) energy intakes at admission and discharge in older inpatients with severely low BMI group were 24.4 (21.0-27.8) kcal/IBW/day and 26.4 (23.6–30.1) kcal/IBW/day, respectively, and these energy intakes did not reach to 35 kcal/IBW/day. In other words, the energy intake of the severely low BMI group was considered to be insufficient. This might have led to no change in body weight during the hospital stay of the older inpatients with severely low BMI.

Nutritional care for increasing body weight in older inpatients with low body weight is essential for improving their ADL [5, 6]. However, the body weight of the older inpatients with severely low BMI did not increase during the hospital stay in this study. In contrast, provided rehabilitation degree and ADL during the hospital stays of the older inpatients with severely low BMI were significantly increased. In other words, the background that the body weight of the older inpatients with severely low BMI did not increase is considered to be attributed to an increase in physical activity (i.e., improvements of provided rehabilitation and ADL) in addition to lack of energy intake. If conducting better appropriate nutritional care, ADL recovery of older inpatients with severely low BMI could have been maximized.

Our study has three limitations. First, this study targeted the quadriceps for muscle mass assessment. Therefore, relationships between changes in other muscle mass and body weight are unclear. Second, the body weight of older inpatients with severely low BMI was not significantly changed in this study. If targeting older inpatients with severely low BMI who have a loss or an increase of body weight, different results may be confirmed. Finally, this study included patients aged ≥ 70 years. Therefore, whether similar results are obtained when target patients aged < 70 years remains unclear.

## Conclusions

The results of this study suggest that change in body weight is positively related to the change in muscle mass of the quadriceps in older inpatients with severely low BMI according to the GLIM criteria, although we targeted the severely low BMI group with no body weight change during the hospital stay. These results imply the importance of body weight management for older inpatients with severely low BMI perspective from the muscle mass of the quadriceps.

## Data Availability

All data generated or analysed during this study are included in this published article.

## Abbreviations

ADL: Activities of daily living
GLIM: Global Leadership Initiative on Malnutrition
BMI: Body mass index
IBW: Ideal body weight
GNRI: Geriatric Nutritional Risk Index
FILS: Food Intake Level Scale
CRP: C-reactive protein
UCCI: Updated Charlson Comorbidity Index
BI: Barthel Index
